# Differential infection outcome of *Chlamydia trachomatis* in human blood monocytes and monocyte-derived dendritic cells

**DOI:** 10.1186/s12866-014-0209-3

**Published:** 2014-08-14

**Authors:** Baishakhi Datta, Florence Njau, Jessica Thalmann, Hermann Haller, Annette D Wagner

**Affiliations:** 1 Department of Nephrology, Hannover Medical School, Hannover, Germany

**Keywords:** Chlamydia trachomatis, Monocyte, Dendritic cell, Cytokine, Gene expression

## Abstract

**Background:**

*Chlamydia trachomatis* is an intracellular bacteria which consist of three biovariants; trachoma (serovars A-C), urogenital (serovars D-K) and lymphogranuloma venereum (L1-L3), causing a wide spectrum of disease in humans. Monocytes are considered to disseminate this pathogen throughout the body while dendritic cells (DCs) play an important role in mediating immune response against bacterial infection. To determine the fate of *C. trachomatis* within human peripheral blood monocytes and monocyte-derived DCs, these two sets of immune cells were infected with serovars Ba, D and L2, representative of the three biovariants of *C. trachomatis*.

**Results:**

Our study revealed that the different serovars primarily infect monocytes and DCs in a comparable fashion, however undergo differential infection outcome, serovar L2 being the only candidate to inflict active infection. Moreover, the *C. trachomatis* serovars Ba and D become persistent in monocytes while the serovars predominantly suffer degradation within DCs. Effects of persistence gene Indoleamine 2, 3-dioxygenase (IDO) was not clearly evident in the differential infection outcome. The heightened levels of inflammatory cytokines secreted by the chlamydial infection in DCs compared to monocytes seemed to be instrumental for this consequence. The immune genes induced in monocytes and DCs against chlamydial infection involves a different set of Toll-like receptors, indicating that distinct intracellular signalling pathways are adopted for immune response.

**Conclusion:**

Our results demonstrate that the host pathogen interaction in chlamydia infection is not only serovar specific but manifests cell specific features, inducing separate immune response cascade in monocytes and DCs.

## Background

*Chlamydia trachomatis* is an obligate intracellular pathogen with unique biphasic developmental cycle alternating between the infectious elementary body (EB) and the metabolically active reticulate body (RB). Based on the antigenic variation of the major outer membrane protein (MOMP), the *C. trachomatis* isolates have been divided into three different biovariants [1]. Serovars A, B, Ba and C cause ocular infections, currently infecting 150 million people worldwide [2],[3]; serovars D, E, F, G, H, I, J and K cause sexually transmitted disease with more than 90 million new cases of genital infections occurring every year [4],[5] and serovars L1, L2 and L3 cause lymphgranuloma venereum (LGV) primarily affecting the lymphatic system with recent outbreaks in Western Europe [6],[7]. Comparative genomic studies demonstrate that the genome of *C. trachomatis* serovars are strikingly similar to each other and share more than 99% identity [8],[9]. Genetic differences were observed centring around the plasticity zone i.e. ~50 kb region near the predicted termination origin of the genome, experiencing a higher degree of DNA arrangement [10], MOMP and members of polymorphic membrane protein (pmp) gene family [11]. However the occurrence of quantitatively different infections by different serovars within a given host has been intriguing. In vivo studies infecting mice intranasally [12] or intravaginally [13] with different serovars of *C. trachomatis* has revealed a great deal of variation in infection kinetics. Genome analysis could reveal that a functional tryptophan synthase enzyme and toxin might be the principal virulence factors underlying this distinct tropism in terms of organ specific disease termed as organotropism [14]. Studies including LGV serovars confirmed the observation that the variability resided mainly in the plasticity zone [15].

Chlamydia primarily targets the host epithelial cells and resides within distinct membrane bound vacuoles termed as chlamydial inclusion. The chlamydia proliferate within inclusion and inhibits their acidification by avoiding fusion with lysosomal compartments [16],[17]. However the association of *C. trachomatis* with reactive arthritis have raised questions how chlamydia is transported from the site of infection to the site of inflammatory disease in the joints or vasculature [18]-[20]. Studies have shown that the *C. trachomatis* infection is characterized by infiltration with polymorphonuclear leukocyte (PMNs) in the acute phase and mononuclear cells in the chronic phase [21]. Hence there have been suggestions that circulating monocytes delivers the pathogen to other organs and initiate immunological response and inflammation. The role of *C. pneumoniae* infected monocytes as a vector, transmitting the pathogen to vascular wall, has been elucidated earlier [22]. The *C. trachomatis* infection of monocytes in vitro, have mostly resulted in noncultivable state in which the bacteria although metabolically active could not produce active infectious particle when recultured in HeLa cells [23],[24].

Dendritic cells (DCs) are the first professional antigen presenting cells encountering the bacteria after initial infection. DCs are very efficient in processing and presenting bacterial antigens and play a crucial role in activating T cell-dependent immune response [25],[26]. Studies have illustrated the role of DCs to evoke strong immune responses against chlamydial infections by stimulating T cell reaction [27],[28]. There are contrasting evidences of the fate of *C. trachomatis* within DCs; there has been observations that *C. trachomatis* inclusion fuses with lysosomal compartment [29] while another study confirmed that the chlamydial inclusion did not colocalize with lysosome associated membrane protein (Lamp) 1 or Major histocompatibility complex (MHC) II compartments [30]. *C. trachomatis* infection of DCs was characterized by up-regulation of co-stimulatory molecules and secretion of inflammatory cytokines [31]. Previous studies have implicated cytokines IFN-γ as well TNF of inducing indoleamine 2,3-dioxygenase (IDO), an enzyme catalysing the degradation of tryptophan leading to chlamydial growth arrest [32]-[34]. The presence of a functional tryptophan synthase in the urogenital serovars while its absence in the ocular serovars [35],[36] has been considered to be pivotal. The genital serovars survive by utilizing indole produced by vaginal microbial flora as a substrate for tryptophan synthesis in IDO induced tryptophan-depleted culture medium [37].

However, little is known about the growth characteristics of the different biovariants of *C. trachomatis* in monocytes and DCs -the two major immune cells that the bacterium encounters during infection. Hence we selected three serovars Ba, D and L2; representative of the ocular, urogenital and lymphogranuloma serovars respectively, for comparative study in human monocytes and monocyte- derived DCs. In our study we observed the chlamydial morphology within infected monocytes and DCs; analyzed their metabolic activity and could illustrate the cytokine induced inflammatory response. We were also able to propose the distinct immune response pathways employed by *C. trachomatis* infected monocytes and DCs.

## Methods

### Chlamydia culture

*Chlamydia trachomatis* serovars D/UW-3/Cx(ATCC-VR885) and serotype LGV II strain 434(ATCC-VR902B) were kindly provided by Prof Andreas Klos (Medical Microbiology and Hospital Epidemiology, Hannover Medical School, Germany) and *Chlamydia trachomatis* serotype Ba Apache-2(ATCC-VR347) was kindly sent by Prof Eberhard Straube (Institute of Medical Microbiology, Friedrich Schiller University of Jena, Jena, Germany).

Bacterial stocks were prepared as described previously [38]. Briefly, HeLa cell monolayers infected with *C. trachomatis* serovar Ba, D and L2 EBs were cultivated at 37°C and 5% CO_2_ in Earle's MEM containing glutamine, supplemented with 10% fetal calf serum (FCS), 0.1 M nonessential amino acids, and 1 mM sodium pyruvate (PAA Laboratories, Pasching, Germany) along with 1 μg/ml cycloheximide (Sigma-Aldrich, Steinheim, Germany). EBs from infected cells were harvested at 48 hours (Serovar L2) to 72 hours (Serovar Ba and Serovar D) p.i., purified by 2 step ultracentrifugation and collected in transport medium (1x PBS, including 6.86% saccharose, 40 μg/ml Gentamicin, 0.002% Phenol red, 2% FCS)*.* The final stock was stored in small aliquots in transport medium at −80°C until use. Mock control was prepared following the complete propagation, harvest and purification procedure for EBs in the absence of *C. trachomatis* infection. All the stocks were free of *Mycoplasma* as tested by Venor GeM kit (Minerva Biolabs, Berlin, Germany). To quantify the EB, the inclusions were counted and the EB determined as inclusion-forming-units (IFU)/ml. For heat inactivation, EBs of *C. trachomatis* serovars Ba, D and L2 were treated at 75°C for 30 minutes. All the plastic wares were obtained from Greiner Bio-One (Greiner Bio-One GmbH, Frickenhausen, Germany) unless otherwise mentioned.

### Culture of monocytes and monocyte-derived DCs

Heparinized buffy coats from healthy blood donors were obtained from Blutspendedienst NSTOB Springe, Bremen, Germany. Buffy coats were prepared from whole blood collected from volunteer donors under informed consent according to the current German hemotherapy guidelines [39]. Peripheral blood mononuclear cells (PBMCs) were isolated by Ficoll-Hypaque density gradient centrifugation using Lymphocyte Separation Medium (PAA Laboratories, Pasching, Germany). For each experiment a different blood donor was used. Monocytes were isolated by negative selection using the Monocyte Isolation kit II (Miltenyi Biotec GmbH, Bergisch Gladbach, Germany) according to manufacturer’s protocol (monocyte purity >90%).

Monocytes were seeded on Poly L-Lysine (0.01%) coated 24-well plate at a density of 3×10^5^, allowed to adhere for 2 hours before infection and cultured in RPMI-1640 (PAA Laboratories, Pasching, Germany) containing 10% FCS. For DCs, 3×10^5^ monocytes were cultured in RPMI-1640 medium with autologous serum in 24-well plate for 7 days in the presence of IL-4 (1000 U/mL) (R&D Systems, Wiesbaden, Germany) and GM-CSF (500 U/mL) (Novartis Pharma, Nurnberg, Germany) as described previously [40].

### Infection of monocyte and monocyte-derived DC

Monocytes and the monocyte-derived DCs were infected with *C. trachomatis* serovars Ba, D and L2 at a multiplicity of infection (MOI) of 3 by centrifugation for 30 min at 400 *× g* with further incubation for 30 min at 37°C in 5% CO_2_. Following incubation, the cells were washed with phosphate-buffered saline (PBS) to remove extracellular bacteria. Fresh RPMI-1640 containing 10% FCS was added to infected monocytes and RPMI-1640 with autologous serum was added to infected DCs. The infected cultures were incubated at 37°C and 5% CO_2_ for intended durations. For immunofluorescence, cells were grown on coverslips. Infected cells were harvested by rubber scraper at different time points as per experimental protocol. The cell pellets for PCR/reinfection as well as supernatants for cytokine analysis were stored at −80°C. Mock infected controls were prepared for every set of experiment to assess the contribution of host cell debris. Control samples were routinely checked for the presence of chlamydia antigens in the donor samples by immunofluorescence microscopy.

### Immunofluorescence microscopy

The infected monocytes and DCs after intended incubation were fixed in 2% para-formaldehyde for 10 min and washed 3 times in PBS. Cells were permeabilized with 0.5% TritonX-100 for 3 minutes. Following fixation, the cells were blocked with PBS containing 1% BSA and 1% FCS. Genus-specific fluorescein isothiocyanate (FITC)-labelled monoclonal antibody (Pathfinder *Chlamydia* Confirmation System; Bio-Rad, Redmond, WA) was used to stain the chlamydial inclusions, while the monocytes and DCs were counterstained with Evan’s Blue at room temperature for 45 min. The samples were then washed once with PBS and then washed twice with PBS/DAPI (1:2500) to stain the cell nuclei. Images were captured in 10 random fields with a fluorescence microscope (Leica DMLB, Germany) with standard filters at 63X magnification. ImageJ was used to count the number of inclusions/cells in replicate samples. Data from 3 independent experiments were combined to calculate the mean and standard deviation.

### Analysis of the infectivity of *C. trachomatis* in monocytes/DCs

Cells harvested at different time points were lysed in an ultrasonic sonicator bath (Jürgen’s Hannover, Germany). Cell lysates were used to infect HeLa cells seeded on coverslips and cultured in MEM media containing 1 μg/ml cycloheximide at 37°C in 5% CO_2_ for the intended duration. At the end of the infection period, cells were fixed for 10 min in absolute methanol, air-dried, and stained using FITC-labelled monoclonal antibody (Pathfinder *Chlamydia* Confirmation System; Bio-Rad, Redmond, WA) and counterstained with Evan’s blue. Images were captured in 10 random fields with a fluorescence microscope (Leica DMLB, Germany) with standard filters at 40X magnification. The inclusions were counted as described under section Immunofluorescence microscopy. Data from 3 independent experiments were combined to calculate the mean and standard deviation.

### Gene expression analysis by real-time PCR

For the analysis of chlamydial gene expression, infected cells were harvested at different time points and real-time PCR was performed targeting the 16S rRNA gene as described previously [34]. To analyse chlamydial developmental phase, expression of genes *euo*, *ompA* and *omcB* were performed. All the primers were ordered from Eurofins MWG Operon (Additional file 1: Figure S1). Briefly, total RNA was isolated from the cell pellets of infected monocytes or DCs using the Macherey Nagel kit (Macherey-Nagel GmbH, Dueren, Germany). 500 nanogram of RNA was reverse-transcribed from each sample using the Eurogentec Reverse-Transcription Kit. Real-time PCR was performed using the qPCR Core kit (Eurogentec) in Roche Lightcycler 480 system. The gene expression levels were calculated by the delta-delta Ct (ddCt) method [41], normalized to 16S in case of chlamydial genes and to 18S for host genes, and compared to the mock sample as the reference gene. The specificity and identity of the amplified products were determined using Light Cycler 480 melting curve analysis software. Data from 3 independent experiments with pool of 2 donors were combined to calculate the mean and standard deviation.

### Quantification of cytokines

The level of the cytokines IL-1β, IL-6, IL-8, IL-10, TNF and IL-12p70 were measured in the supernatants of the infected monocytes and DCs collected 1 day p.i. by Cytometric Bead Array (Human Inflammatory Cytokines Kit; BD Biosciences, San Diego, CA) according to the manufacturer's instruction. In brief, 50 μL of human inflammation capture bead suspension and 50 μL of phycoerythrin detection reagent were added to an equal amount of samples or standard dilution and incubated for 3 hours at room temperature in the dark. The monocyte samples were diluted 1:2 and DCs samples were diluted 1:4 with assay diluent to have sample data within the range of the standard curve. Subsequently, samples were washed with wash buffer and centrifuged at 200 × *g* at room temperature for 5 minutes. The samples were further fixed with 2% paraformaldehyde for 30 minutes. The supernatant was discarded and 300 μL of wash buffer was added. Samples were then analysed on a BD FACS Calibur flow cytometer (BD Biosciences, Heidelberg, Germany). The data was analyzed using the FCAP array software (BD Biosciences). Data from 3 independent experiments with pool of 2 donors were combined to calculate the mean and standard deviation.

### Innate and adaptive immune response array

The Human Innate and Adaptive Immune response Array (PAHS-052) was performed using the SYBR green-based RT2 Profiler system (SA Biosciences, Frederick, MD). This PCR array is a pathway focused array that contains a set of 84 related genes involved in the inflammatory immune response. This assay also contains 5 housekeeping genes and 3 other reaction controls to assess genomic DNA contamination, RNA quality, and general PCR performance. Total RNA from infected monocytes and DCs were extracted using Macherey Nagel kit (Macherey-Nagel GmbH, Dueren, Germany). Due to the low RNA concentration, monocyte RNA sample were amplified by RT^2^ PreAmp PCR master mix (SA Biosciences, Frederick, MD). Equal amount of RNA from each sample was reverse-transcribed to cDNA by using Reverse-transcription mix preceded by a genomic DNA elimination step; both provided in the kit. The amplification of the immune genes in the samples was investigated by real-time PCR on Roche Light Cycler 480 according to the manufacturer's instructions. Data analysis was performed using manufacturer’s program and is based on the ddCt method, with normalization of the raw data to the panel of housekeeping genes provided in the array. The genes showing modulation by 1.5 fold up or down were only selected for further analysis. Functional annotations of the selected genes were carried out by the bioinformatics software David for Bioinformatics. Three independent experiments with a pool of 2 donors each were analyzed.

### Statistical analysis

Statistical evaluation of the data was done using GraphPad Prism 5 software. Student t-test was performed for simple comparison between 2 means. For multiple comparisons, the results were analysed by two-way ANOVA followed by Bonferoni’s post-test. p < 0.05 was considered statistically significant. All shown data are representative for at least 3 independent experiments.

## Results

### *Chlamydia trachomatis* infect monocytes and monocyte-derived DCs in a comparable manner

**Figure 1:**
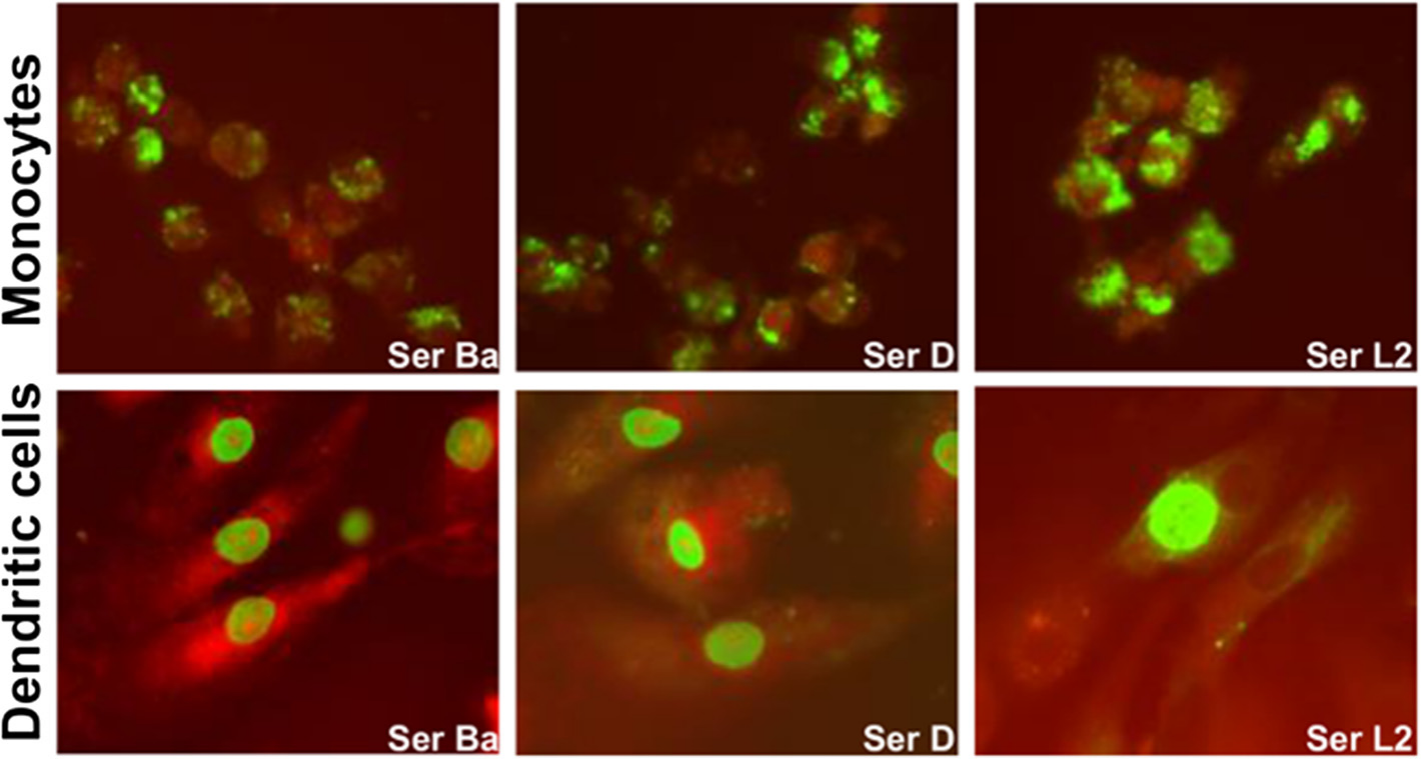
**Immunofluorescence microscopy of infected monocytes and monocyte-derived Dendritic cells (DCs).** Monocytes (upper panel) and monocyte-derived DCs (lower panel) were infected with *C. trachomatis* serovars Ba, D and L2 (MOI-3) for 2 days. Chlamydial inclusions (green) were stained with FITC conjugated anti-chlamydia LPS antibody and counterstained with Evans Blue. Pictures were taken at 63X magnification with Leica DMLB. The figures are representative of 3 independent experiments.

Monocytes isolated from human peripheral blood mononuclear cells (PBMCs) and monocyte-derived DCs were infected with *C. trachomatis* serovars Ba, D and L2 (Figure 1). Results show that all the three serovars were capable of infecting both the monocytes and DCs and form inclusions as detected by immunofluorescence microscopy 2 days post infection (p.i.). However, the inclusions were smaller in size compared to typical inclusions that have been reported in HeLa cells (Additional file 2: Figure S2). The inclusion morphology and staining intensity varied between the infected monocytes and DCs.

In monocytes, the percentage of infected cells were comparable among the three serovars and did not seem to change even when the infection duration was extended to 3 days (Table 1). For DCs, the percentage of infected cells were similar for serovars Ba and D but serovar L2 showed a higher infection rate as compared to the other two (Table 1). However, the infection rate declined remarkably for all the three serovars when infected for 3 days. The infection rate was nevertheless much lower in both monocytes and DCs than in HeLa. Mock controls were prepared for each round of experiments which showed absence of chlamydial antigens in the donors (Additional file 3: Figure S3).

**Table 1:** **Comparison of infection rate in monocytes and monocyte-derived DCs infected with** ***C. trachomatis*** **serovars Ba, D and L2**

**Infection percentage (%)**						
	**Serovar Ba**		**Serovar D**		**Serovar L2**	
	**2 days p.i**	**3 days p.i**	**2 days p.i**	**3 days p.i**	**2 days p.i**	**3 days p.i**
**Monocytes**	32 ± 5	34.3 ± 6	33.6 ± 6	36.6 ± 7	44 ± 6	42 ± 3
**DC**	26.8 ± 2	20.7 ± 2	29.4 ± 1	24.4 ± 1	39.9 ± 4	25.4 ± 2
**HeLa**	78 ± 7	81.3 ± 6	83.5 ± 4	85.1 ± 7	88.7 ± 3	84.2 ± 3

Monocytes, DCs and HeLa cells were infected with *Chlamydia trachomatis* serovars Ba, D and L2 and stained with anti-Chlamydia LPS antibody at 2 day and 3 day p.i.. Quantification of chlamydia infected cells were done by counting total number of cells (indicated by nuclei staining) and cells positive for Chlamydia and from 15 pictures The mean and ± SD were calculated from three independent experiments.

### Differential development of *C. trachomatis* serovar L2 in monocytes and DCs

In our study, we further investigated the survival and re-infection potential of chlamydia serovars after the primary infection of monocytes and DCs. Chlamydia-infected monocytes and DCs were harvested 2 days p.i. and passaged onto HeLa cell confluent monolayer. HeLa cells were investigated by immunofluorescence microscopy 2 days p.i. and the inclusions were counted.

**Figure 2:**
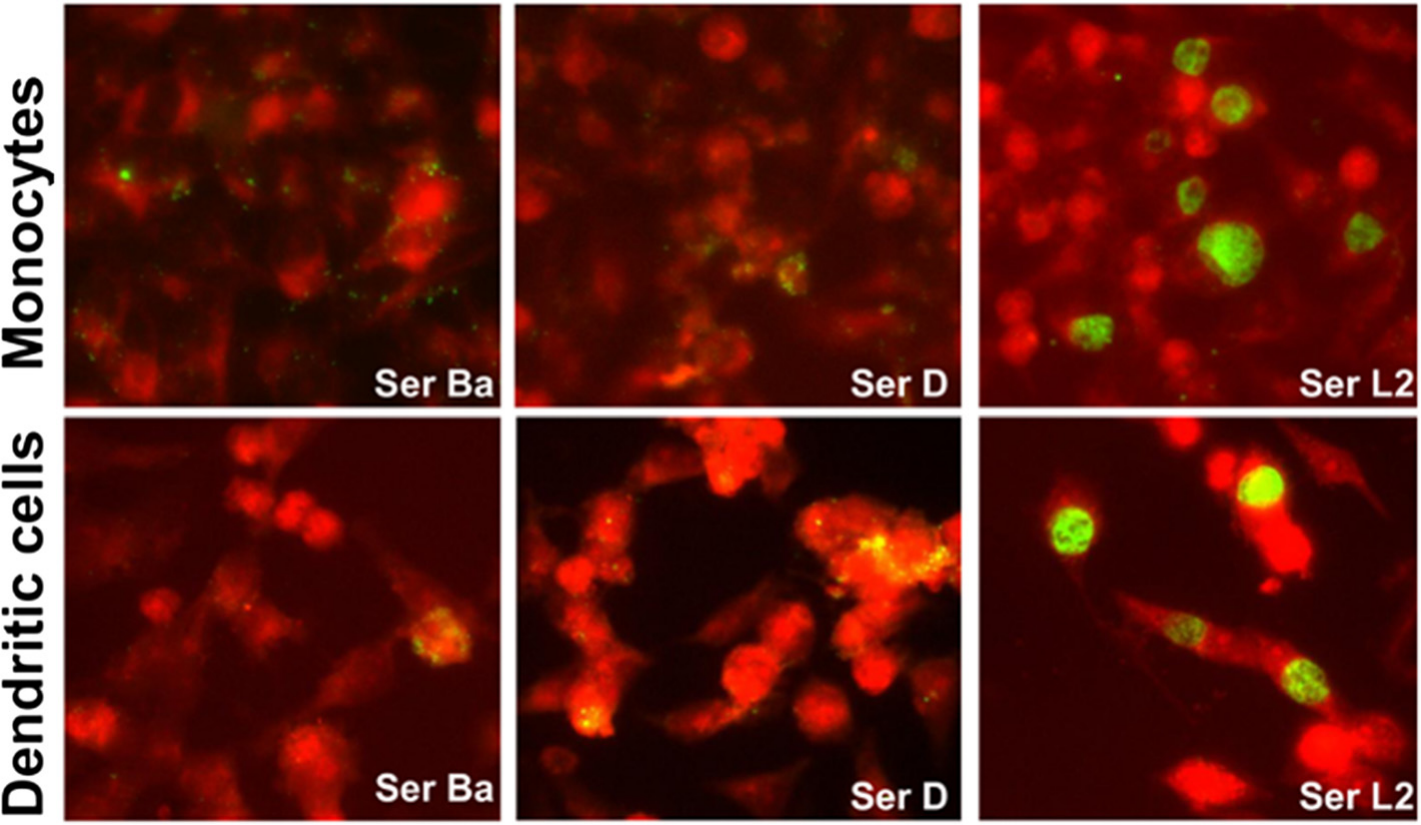
**Infectivity assay of Chlamydiae infected monocytes and monocyte-derived DCs.** Monocytes (upper panel) and human monocyte-derived DCs (lower panel) were infected with *C. trachomatis* serovars Ba, D and L2 (MOI-3) for 2 days and were further passaged in HeLa cells for 2 days. Chlamydial inclusions (green) were stained with FITC conjugated anti-chlamydia LPS antibody and counterstained with Evans Blue. Pictures taken at 40X magnification with Leica DMLB. The figures are representative of 3 independent experiments.

The serovars Ba and the D were not able to produce inclusions in HeLa cells after infecting either monocytes or DCs for 2 days. Only scattered antigens could be detected (Figure 2). Interestingly, serovar L2 produced inclusions in HeLa cells after infecting both monocytes and DCs (Figure 2). There was no recovery of infectious progeny from serovars Ba and D even with longer duration of primary infection (3 days) or if the passage in HeLa cells was carried out for a longer duration (72 hours) (data not shown). In the case of serovar L2, passaging for longer time did not yield a higher number of infectious progeny.

### Metabolic activity of chlamydia within infected monocytes and DCs

To characterize the metabolic activity of chlamydiae in monocytes and DCs, we investigated the expression of 16S rRNA gene transcripts which reflects the growth rate and/or metabolic activity of chlamydiae in the cells [40]. The expression of 16S rRNA in chlamydiae-infected monocytes and DCs was assessed over 3 days after infection.

**Figure 3:**
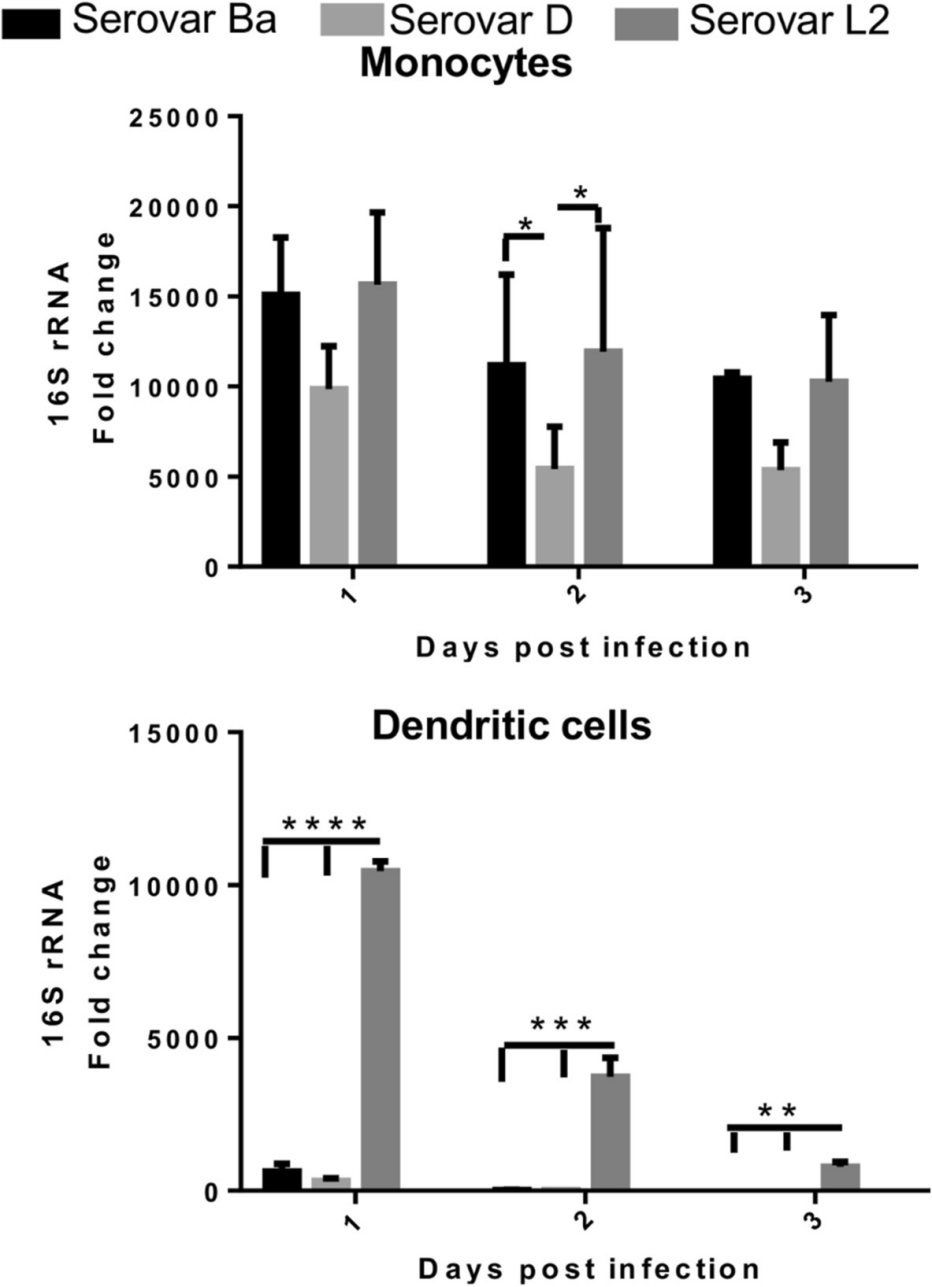
**Metabolic activity of intracellular chlamydiae in infected monocytes and monocyte-derived DCs.** Monocytes and monocyte-derived DCs were infected with *C. trachomatis* serovars Ba, D and L2 (MOI-3) and mock control. 16S rRNA gene copy numbers was determined by isolating RNA at the indicated time points, followed by real-time PCR as described in materials and methods. 16S rRNA fold change was normalized to 18S rRNA and determined by ddCt method with mock sample as reference gene. The mean of 3 independent experiments is shown and each experiment is pool of 2 donors. ***P < 0.001, **P < 0.01, *P < 0.05.

16S rRNA was highly expressed in the infected monocytes for all three chlamydia serovars Ba, D and L2 throughout the 3 day time course of infection (Figure 3). The heightened metabolic activity of the intracellular chlamydia did not seem to decline with a longer duration, thus indicating that all the serovars were viable and metabolically active within the monocytes.

In contrast 16S rRNA expression level was negligible in DCs for serovars Ba and D at 1 day p.i. and further declined with infection progression (Figure 3). Serovar L2 displayed highly significant expression of 16S rRNA at 1and 2 day p.i. Although the level declined on the 3 day p.i., the expression remained significant (Figure 3).

**Figure 4:**
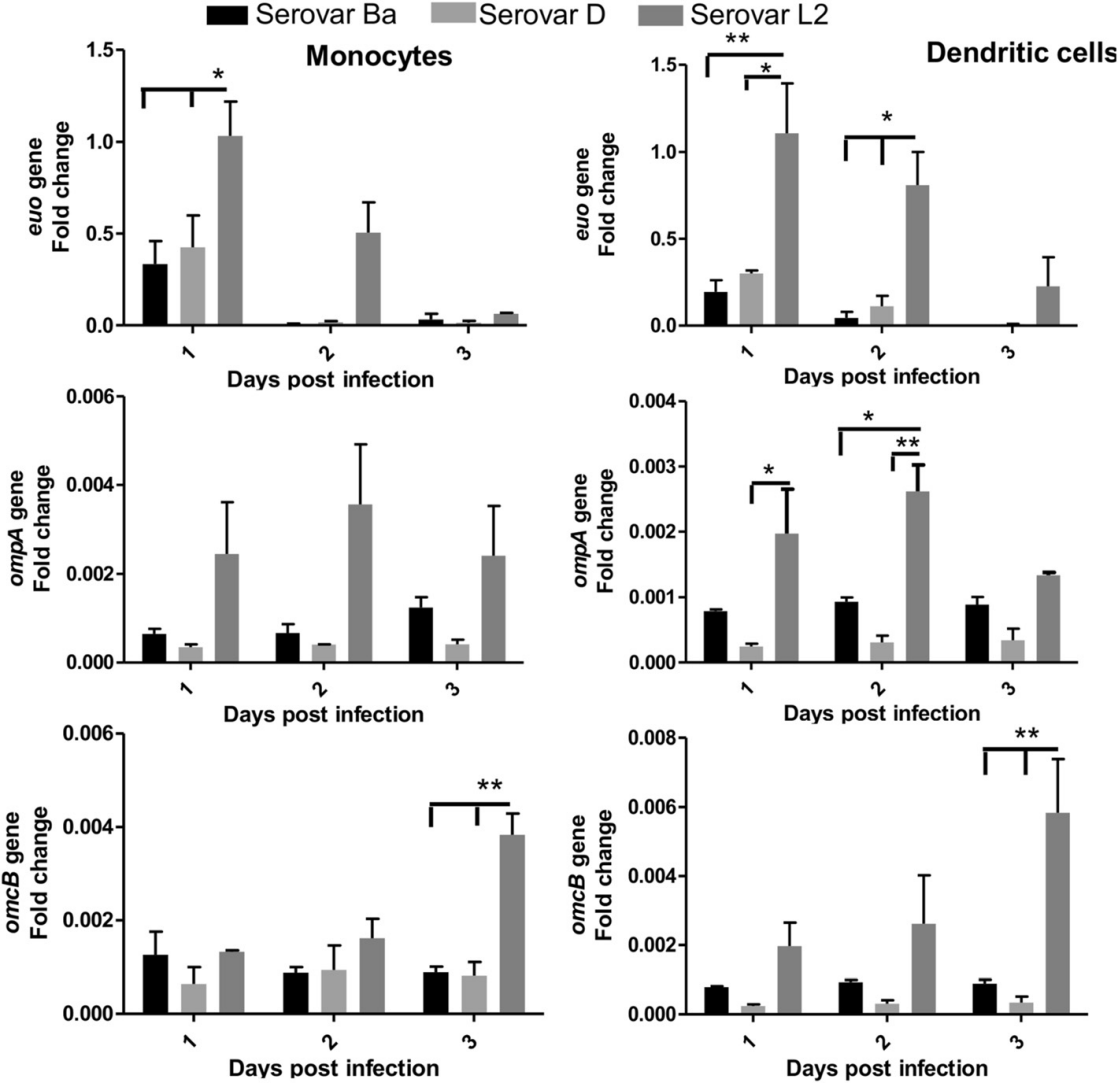
**Quantification of*euo*,*ompA*and*omcB*gene expression in chlamydiae infected monocytes and monocyte-derived DCs.** Monocytes and monocyte-derived DCs were infected with *C. trachomatis* serovars Ba, D and L2 (MOI-3) and mock control. Copy numbers of *euo*, *ompA* and *omcB* genes were determined by isolating RNA at the indicated time points, followed by real-time PCR as described in materials and methods. Gene fold change was normalized to chlamydial 16S rRNA and determined by ddCt method with mock sample as reference gene. The mean of 3 independent experiments is shown and each experiment is pool of 2 donors. ***P < 0.001, **P < 0.01, *P < 0.05.

To further characterize developmental state of chlamydial serovars within the infected monocytes and DCs, gene expression of *euo, ompA* and *omcB* were investigated. Each of these genes are known to be expressed at different developmental stages of chlamydiae (early, mid and late phase respectively), and have previously reported to be transcriptionally altered during chlamydial growth in human monocytes and DCs [40],[42]. Figure 4 depicts the expression of the three genes in monocytes and DCs respectively. Expression of the 3 genes within serovars Ba and D in both cell types was similar and stable, albeit at low levels in all the three time points that were investigated. Serovar L2 depicted a different pattern; early stage gene *euo* was significantly expressed 1 day p.i. compared to serovars Ba and D, gradually diminishing with time in both monocytes and DCs. The expression of mid-cycle gene *ompA* for serovar L2, although higher than the serovars Ba and D, was not statistically significant in infected monocytes. The expression for *ompA* within infected DCs peaked at 2 day p.i. significant to both serovars Ba and D. Expression of late stage gene *omcB* increased significantly 3 days p.i. for serovar L2 compared to serovars Ba and D in both monocytes and DCs.

### Indolamine 2, 3-dioxygenase expression is down-regulated in monocytes and upregulated in DCs by *C. trachomatis* serovar L2

One aspect of chlamydial infection is the gamma-interferon (IFN-γ) mediated induction of Indolamine-2, 3-dioxygenase (IDO), an enzyme catabolizing breakdown of tryptophan in culture media. The unavailability of this essential amino acid can lead to chlamydial growth arrest termed as persistence [43]. The role of TNF-α mediated IDO induction in DCs [44] as well IFN-γ independent IDO activation in monocytic THP-1 cells have been reported earlier [45]. We considered that the level of IDO gene expression could be crucial in understanding the contrasting infection outcome by the chlamydia serovars in monocytes and monocyte-derived DCs. Hence the expression of IDO gene in chlamydiae-infected monocytes and DCs was detected over 3 days post infection.

**Figure 5:**
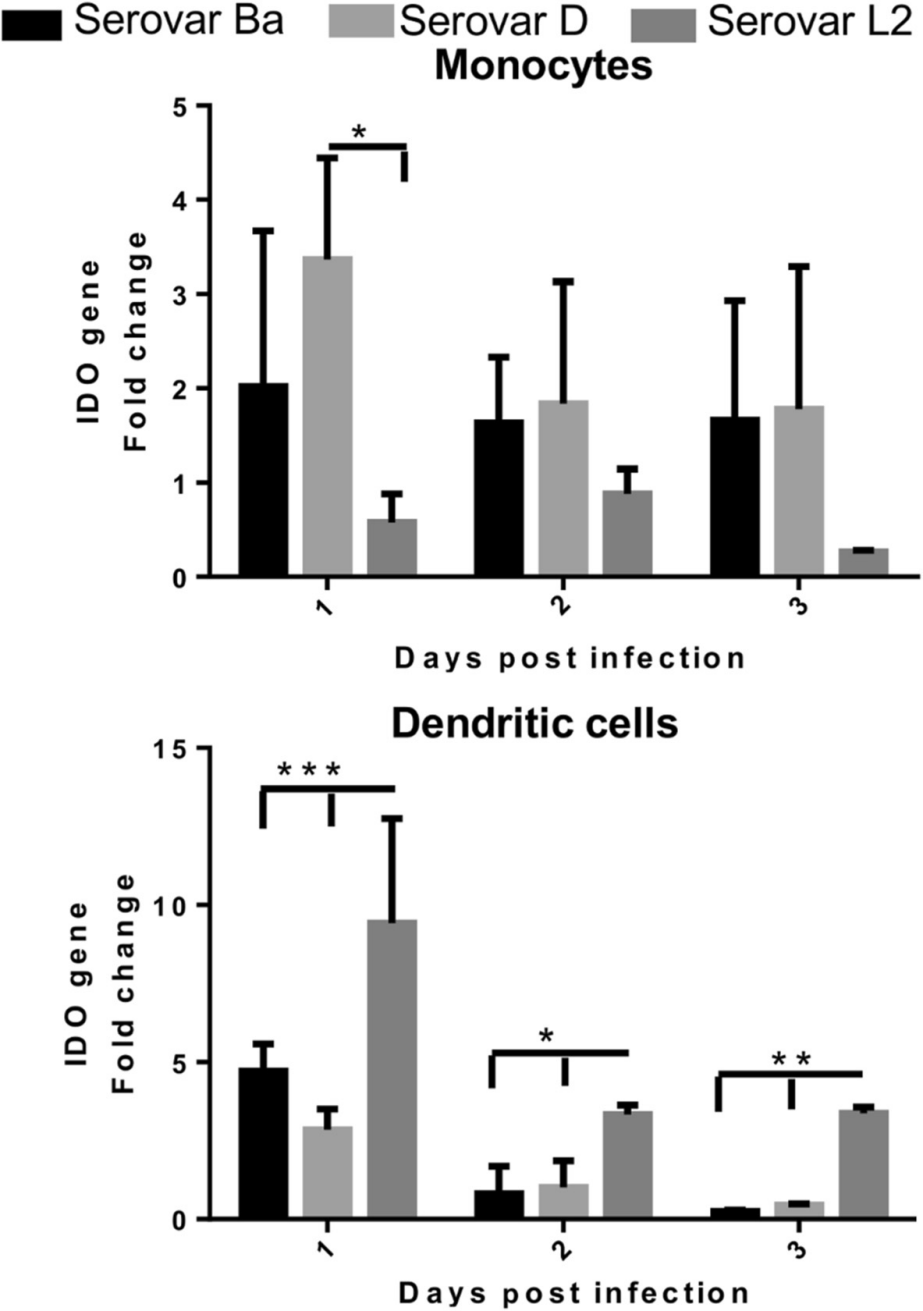
**Indolamine 2, 3- dioxygenase (IDO) gene regulation in chlamydiae-infected monocytes and DCs.** Monocytes and monocyte-derived DCs were infected with *C. trachomatis* serovars Ba, D and L2 (MOI-3) and mock control. IDO gene copy numbers was determined by isolating RNA at the indicated time points followed by real-time PCR as described in materials and methods. IDO fold change was normalized to 18S rRNA and determined by ddCt method with mock sample as reference gene. The mean of 3 independent experiments is shown and each experiment is pool of 2 donors. ***P < 0.001, **P < 0.01, *P < 0.05.

Monocytes, infected with serovars Ba and D expressed higher levels of IDO 1 day post infection (Figure 5). Contrastingly, IDO expression by serovar L2 infected monocytes was significantly down-regulated 1 day p.i compared to serovar D. On the other days of infection the trend was similar but not significant.

IDO expression was significantly up-regulated in DCs infected with serovar L2 (Figure 5) compared to serovars Ba and D. IDO expression declined throughout the infection course for all the servers, however maintaining a significant expression for serovar L2 infection.

Attempts were made to enhance chlamydial recovery from infected monocytes and DCs by addition of Tryptophan, known to be depleted by IDO during chlamydial infection [34],[46]. However the infected cultures supplemented with Tryptophan (200 μg/ml) when passaged on HeLa cells could not abrogate the growth arrest; chlamydial inclusions could not be recovered from serovar Ba and D cultures (data not shown). However, Serovar L2 could produce chlamydial inclusions irrespective of Tryptophan.

### Differential cytokine response induced in monocytes and DCs by chlamydial infection

We investigated the role of cytokines in mediating contrasting infection outcome of chlamydia infection the monocytes and DCs. Supernatants were collected from monocyte and monocyte-derived DCs culture infected with *C. trachomatis* serovars Ba, D and L2 at 1 day p.i. and cytokine responses were assessed by Cytokine Bead Array.

**Figure 6:**
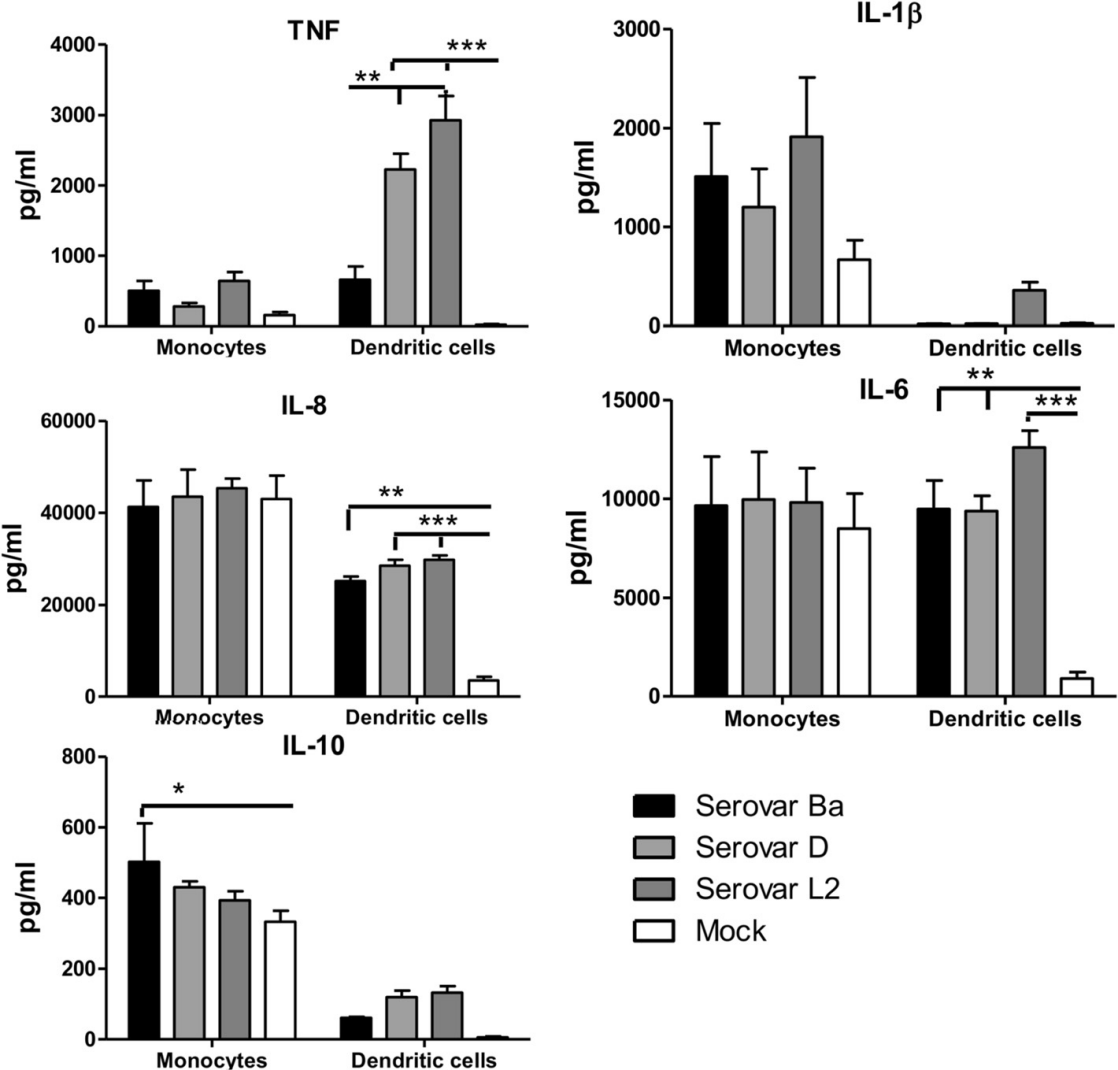
**Cytokine concentration in chlamydiae-infected monocytes and monocyte-derived DCs.** Monocytes and monocyte-derived DCs were infected with *C. trachomatis* serovars Ba, D and L2 (MOI-3) and mock control. Supernatants were collected 1 day post infection and the concentration of the different cytokines IL-1β, TNF, IL-6, IL-8 and IL-10 were determined by using the kit Cytometric Bead Array. The concentration is reported as pg/ml. The cytokine secreted by heat-killed sample of each serovar were quantified and are indicated for each dataset. The mean of 3 independent experiments is shown and each experiment is pool of 2 donors. ***P < 0.001, **P < 0.01, *P < 0.05.

As depicted in Figure 6, infection of monocytes and monocyte-derived DCs with chlamydial serovars Ba, D and L2 could induce detectable secretion of cytokines IL-8, IL-6, IL-1β, IL-10 and TNF. Production of IL-12p70 was below the standards (data not shown).

Pro-inflammatory cytokines IL-1β and TNF was elevated in the chlamydiae infected monocytes than the mock control, however were not statistically significant. The level of cytokines IL-6 and IL-8 in infected monocytes showed no statistical difference with mock control. The anti-inflammatory cytokine IL-10 was induced in higher levels than the mock with serovar Ba infection secreting significant amounts compared to mock. DCs infected with serovars D and L2 showed significantly up-regulated levels of TNF. The other pro-inflammatory cytokine IL-1β although secreted in higher amounts within serovar L2 infected DCs, than the other serovars or mock, was not significant. DCs infection resulted in significant production of inflammatory cytokines IL-8 and IL-6. The anti-inflammatory cytokine IL-10 levels were low in the infected DCs and were not statistically significant to the mock control. To understand LPS contribution in the observed cytokine responses, monocytes and DCs were infected with heat-killed *C. trachomatis* serovars Ba, D and L2 EBs at MOI-3 and the cytokine levels were investigated (Additional file 4: Figure S4). Heat-killed EBs for serovar Ba and D induced significantly low level of IL-8 and IL-6 in monocytes while the TNF levels were low in DCs for serovar D and L2. The most remarkable observation was the negligible induction of IL-10 by heat-killed EBs from all 3 serovars in monocytes which was highly significant.

### Immune gene response to *C. trachomatis* infected monocytes and DCs

**Figure 7:**
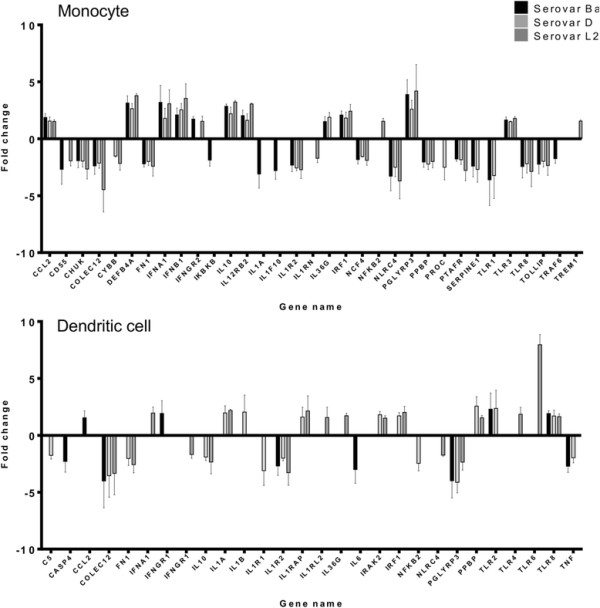
**Genes up-regulated or down-regulated in response to*C. trachomatis*infection in monocytes and DCs.** Expression of Innate and adaptive immune response genes were studied by PCR array in monocytes and DCs infected with *Chlamydia trachomatis* serovars Ba, D and L2. RNA samples were transcribed into cDNA and expression studied by real-time PCR. The change in fold was studied by ddCt method and genes regulated 1.5 fold up or below the mock control are only included. The mean of 3 independent experiments is shown and each experiment is pool of 2 donors.

To determine the host genes activated by chlamydia infection, the immune response was analyzed by Human innate and Adaptive Immune response array. Genes differentially regulated 1.5 fold up or down in monocytes or monocyte-derived DCs infected with *C. trachomatis* serovars Ba, D and L2 24 hours p.i. were considered for further analysis (Figure 7).

As depicted in Figure 7, Serovar Ba induced up regulation of 11 genes, Serovar D of 11 genes and serovar L2 of 13 genes within infected monocytes. Of these up-regulated genes 8 genes were common in all 3 serovars which included receptor for bacterial components (PGLYRP3) and genes responsible for antibacterial defense (DEF4BA, CCL2). Cytokine genes inducing antiviral effect (IFNA1, IFNB1) as well as immune-regulation (IL-10) were also elevated emphasizing the cytokine interplay in infected monocyte. It is noteworthy that Toll-like receptor (TLR) 3 which recognizes dsRNA and is crucial for the TRIF mediated immune response pathway (MyD88 independent) was up-regulated. TREM1 gene, which is an important sepsis marker, was elevated in serovar L2 infected monocytes.

The down-regulated genes in the infected monocytes numbered 19 for serovar Ba, 15 for serovar D and 14 for serovar L2 (Figure 7). Ten of those genes were common for all the 3 serovars which included a member of Myd88 dependent pathway (TLR8) and interacting protein (TOLLIP). Other genes involved were predominantly involved in vascular mechanism (PTAFR, PPBP, FN1 and COLEC12). Additionally, some genes involved in apoptosis and oxidative process (CHUK, NCF4 and NLRC4) were also down-regulated.

DCs response to the chlamydial serovars were also intriguing. There was up regulation of 4 genes by serovar Ba, 7 genes by serovar D and 10 genes by serovar L2 (Figure 7). The remarkable observation was that serovars Ba, D and L2 could all up regulate TLR8 as well other TLRs individually (TLR, 2, 4 and 6), all belonging to the Myd88 dependent signalling pathway [47].

The genes down regulated in DCs in response to chlamydial infection numbered 4 for serovar Ba, 5 for serovar D and 5 for serovar L2. Two genes were common which included anti-inflammatory effector (IL-10) as well as gene involved in vascular process (COLEC12).

## Discussion

In our study we could demonstrate that the different serovars of *C. trachomatis* experience altered fate in monocytes and DCs by virtue of the variable host immune response induced by infection. Monocytes and DCs could be primarily infected by *C. trachomatis* serovars Ba, D and L2 in comparable degree. This is in agreement with previous study showing similar results in terms of primary infection of DCs by *C. trachomatis*[31]. To our knowledge, no such study has been reported for monocytes, hence we report here for the first time characteristics of *C. trachomatis* serovars Ba, D and L2 infection in monocytes. The infection percentages were comparable for serovar Ba and D while serovar L2 experienced a slightly higher rate in both monocytes and DCs infection. Incidentally the percentage declined in infected DCs with longer culture period for all the three serovars. The variable inclusion morphology and lower infection rate within chlamydiae-infected monocytes and DCs as opposed to in HeLa indicated that replication cycle of *C. trachomatis* was somewhat restricted in these two immune cell types.

Attempts to recover infectious particles from the monocytes and DCs infected with the chlamydia serovars led to an interesting observation. Monocytes infected with serovars Ba and D when passaged to HeLa cells could not produce any inclusions, which is in accordance with earlier studies demonstrating inability of serovar K to productively infect monocytes [23],[24]. In contrast to earlier observations [48] where mononuclear cells were considered to be microbicidal for all *C. trachomatis* serovars our results revealed that serovar L2 could productively infect monocytes.

A similar trend was observed in DCs, where serovar Ba and serovar D showed abortive infection with no typical inclusions. Infectious particles could only be recovered from serovar L2 infected DCs as has been reported previously by Gervassi *et al.*[31]. However Gervassi *et al.* showed that serovar E passaged in DCs could be further propagated in HeLa cell whereas in our study serovar D, member of the same biovariant could not be propagated in HeLa cells. The differences between these findings can be caused by effects of genetic human host polymorphism (source of DCs are different), differences in culture condition (10% FCS vs 10% autologous serum), or use of different MOIs 10 vs 3).

In conjunction with the reinfection data, high expression levels of 16S rRNA within monocytes infected with serovars Ba, D and L2 indicated that *C. trachomatis* serovars were viable throughout the infection period, even though infectious progeny could only be recovered from L2 infected monocytes. This phenomenon of viability without producing infectious bodies is known as chlamydial persistence [18],[49]. Serovar K infection of monocytes resulted in attenuation of new EB production although genes involved in chlamydial DNA replication were expressed during persistence [20]. Nevertheless our study establishes that this is a general phenomenon occurring in monocytes for several serovars of *C. trachomatis* biovariants. Contrasting observations are provided by DCs infected with the chlamydial serovars. The absence of recovered infectious progeny along with the negligible expression of 16S rRNA in serovars Ba and D in infected DCs 2 days p.i. suggest the loss of metabolic activity of *C. trachomatis* serovars within DCs. This loss of metabolic activity of *C. trachomatis* serovars within DCs indicated towards a probable degradation of chlamydiae. Serovar L2 could however, produce inclusions during reinfection studies and express 16S rRNA 2 days p.i. in DCs but suffered rapid decline in viability 3 days p.i.. DCs have shown the ability to degrade *Chlamydia psittac*i and *C. trachomatis* MoPn [29] but other DC- *C.trachomatis* studies have shown contrasting results, where they showed that *C. trachomatis* serovars were confined within specific vacuoles within DCs being able to replicate [30],[31]. Our results were in contrast to *Chlamydia pneumoniae* infected DCs showing an increase in 16S rRNA expression when infected for 3 days [34]. The study of the chlamydial developmental cycle within the monocytes and DCs by expression of stage-specific genes showed a clear prominence of serovar L2 compared to serovars Ba and D. The observed gene expression for serovar L2 was in accordance with the expected early, mid and late phase patterns and therefore indicative of presence of viable chlamydiae. The difference in gene expression between serovar L2 and the serovars Ba and D indicates the infection severity. The expression of *ompA* and *omcB* genes for serovars Ba and D, within monocytes and DCs, at later time points indicate that some chlamydiae were still viable. While in monocytes these chlamydia were in persistent form, it is possible that in DCs transient level of *C. trachomatis* development is allowed while predominantly inhibiting or degrading the pathogen as it has been reported previously for other monocytic cells [50].

The presence of a functional tryptophan synthase gene in urogenital serovars and the absence of it in ocular serovars has been related with tissue tropism [35],[37]. The tryptophan synthase gene enables the bacteria to use indole as a substrate for tryptophan synthesis when the intracellular tryptophan is depleted by IDO induction during chlamydial infection. In this study, we have shown that IDO expression levels for ocular serovar Ba and urogenital serovar D were similar while LGV serovar L2 showed down-regulation in infected monocytes. In infected DCs, IDO expression was significantly up-regulated for serovar L2 but declined rapidly in the other two serovars. The involvement of TNF secreted by DCs (Figure 6) seemed to be crucial in the up-regulation of IDO, as TNF has been earlier reported to activate IDO expression in human DCs [51]. The heightened level of IDO in serovar L2 could not restrict its active infection probably due to the presence of functional tryptophan synthase in genital serovars as discussed above. IDO expression revealed analogous pattern for serovars Ba and D in both monocytes and DCs which poses a query whether the organotropism is less pronounced within the immune cells.

In infected monocytes the pro-inflammatory cytokines TNF and IL-1β were secreted in higher levels than mock which might be the reason for the restricted chlamydial growth observed, higher secretion of these cytokines has also been reported previously [45]. The significance of TNF in serovar D and L2 infected DCs confirmed their role in restricting chlamydial growth. The inflammatory cytokines IL-8 and IL-6 although secreted in higher levels by the infected monocytes were not significant. One probable reason for such high volumes could be due to the monocyte adherence for 2 hours prior infection which might have induced some activation. The fact that IL-10 was highly induced by serovars Ba, D and L2 within monocytes demonstrates the critical role played by the anti-inflammatory process to prevent degradation of chlamydia and remain viable within the monocytes. DC infection with serovars Ba, D and L2 could induce significant levels of inflammatory cytokines IL-6 and IL-8. The anti-inflammatory IL-10 was secreted in low levels by the serovars, thus displaying dominance of the inflammatory process in DC infection. The distinct interplay of pro-inflammatory and anti-inflammatory cytokines seemed to play role in infection outcome within monocytes and DCs. The cytokine studies with heat-killed EBs showed that TNF was induced by active infection of DCs by serovars D and L2. Infection by viable chlamydia could only induce secretion of IL-10 in monocytes, indicating that an active infection is essential for inducing these particular cytokines in monocytes or DCs. The data demonstrated that monocytes and DCs induce altered levels of cytokines in response to chlamydial infection, and DCs demonstrate a stronger inflammatory role than the monocytes.

Our data manifested distinct activation profiles of immune genes in monocytes and DCs during *C. trachomatis* infection. Although, the fold-regulation was not significant, the differential regulation of the different genes when analysed through functional annotation tool, David for Bioinformatics, could reveal an interesting pattern. The hallmark of this response was the involvement of the Toll like receptor (TLR) signalling pathway-critical mediators of innate immune response recognizing different microbial components [52]-[54]. On contact with their ligands, TLRs engage different adapter molecules to propagate the downstream signalling. The adapter molecule MyD88 is used by all the TLRs (except TLR3) to activate the transcriptional activator NF-κB and induce secretion of TNF, IL-6 and other inflammatory cytokines thus forming the MyD88 dependent pathway [47],[55]. The other pathway recruits TRIF adapter molecule to induce IFNβ and late induction of NF-κB constituting the MyD88 independent pathway [47],[56]. TLR3 is able to signal exclusively through MyD88-independent pathway [57]. The involvement of TLR2 and TLR4 in *C. trachomatis* mediated infection response has been reported by earlier studies [58],[59]. In our studies the up-regulation of TLR3, IFNA1, IFNB1 for serovars Ba, D and L2 in infected monocytes and the simultaneous down regulation of TLR1, TLR8 suggests the dominance of the TRIF mediated signalling in *C. trachomatis* infected monocytes. The converse could be seen in *C. trachomatis* infected DCs where TLR8 was up-regulated for all the serovars and TLR/2/4/6 of MyD88 signalling pathway were differentially up-regulated for the different serovars. With the array findings, one could speculate that two distinct immune response pathways are employed by monocytes and DCs when infected with specific chlamydial serovars. The results could be further studied in detail to ascertain the role played by the different adapter mediated signalling pathways in chlamydia infection.

## Conclusion

Our study demonstrated that *C. trachomatis* serovars Ba, D and L2 infected monocytes and DCs in a comparable manner; however, they underwent differential infection consequences. Serovars Ba and D became persistent in monocytes while they degraded within DCs. Serovar L2 could, however, maintain the development cycle in both monocytes and DCs, although the process was severely impaired. The heightened levels of inflammatory cytokines secreted by the chlamydial infection in DCs compared to monocytes could be instrumental to the differences observed. The host immune genes response to infection displayed distinct activation profile within monocytes and DCs. Collectively, we could establish that the host pathogen interaction in chlamydia infection is not only serovar specific but also cell specific.

## Abbreviations

*C.trachomatis*: *Chlamydia trachomatis*

EB: Elementary body

DC: Dendritic cell

MOI: Multiplicity of infection

p.i.: Post infection

IL: Interlukein

## Competing interests

The authors declare that they have no competing interests.

## Authors’ contribution

BD performed the experiments, acquired, analyzed and interpreted the data, and drafted the manuscript. FN and ADW: made substantial contributions to the conception and design of experiments, interpretation of results, and drafted and critically revised the manuscript. JT and HH made substantial contributions to the conception and design of experiments. All authors read and approved the final manuscript.

## Additional files

## Supplementary Material

Additional file 1: Figure S1. Gene specific primers used for quantitative real-time PCR. Click here for file

Additional file 2: Figure S2. Immunofluorescence microscopy of HeLa cells: HeLa cells were infected with *C. trachomatis* serovars Ba, D and L2 (MOI-3) for 2 days as positive control. Chlamydial inclusions (green) were stained with FITC conjugated anti-chlamydia LPS antibody and counterstained with Evans Blue. Pictures were taken at 40X magnification with Leica DMLB. The figures are representative of 3 independent experiments. Click here for file

Additional file 3: Figure S3. Immunofluorescence microscopy of mock-infected monocytes and monocyte-derived DCs: Monocytes and monocyte-derived DCs were infected with mock control for 2 days. Chlamydial inclusions (green) were stained with FITC conjugated anti-chlamydia LPS antibody and counterstained with Evans Blue. Pictures were taken at 40X magnification with Leica DMLB. The figures are representative of 3 independent experiments. Click here for file

Additional file 4: Figure S4. Effect of heat-killed chlamydia in cytokine induction within infected monocytes and monocyte-derived DCs: Monocytes and monocyte-derived DCs were infected with live and heat-killed EBs of *C. trachomatis* serovars Ba, D and L2 (MOI-3) and mock control. Supernatants were collected 1 day post infection and the concentration of the different cytokines IL-1β, TNF, IL-6, IL-8 and IL-10 were determined by using the kit Cytometric Bead Array. The concentration is reported as pg/ml. The mean of 3 independent experiments is shown and each experiment is pool of 2 donors. ***P < 0.001, **P < 0.01, *P < 0.05. Click here for file
