# Supplementary material for: Differential infection outcome of Chlamydia trachomatis in human blood monocytes and monocyte-derived dendritic cells
Source: BMC Microbiol. 2014 Aug 14;14:209. doi: 10.1186/s12866-014-0209-3 (PMC4236547; doi:10.1186/s12866-014-0209-3)
Supplement: Additional file 1: Figure S1. — Gene specific primers used for quantitative real-time PCR. [file s12866-014-0209-3-S1.doc]

| **Gene** | **Forward primer (5′-3′)** | **Reverse primer (5′-3′)** |
| --- | --- | --- |
| 16S rRNA | GGACCTTACCTGGGTTTGACATG | GCAGCACCTGTGTATATGTCCT |
| *euo* | CGAAGACTACTCGTTGGGAA | CATAGCACCAATGCGTGTAG |
| *ompA* | TGAACCAAGCCTTATGATCGACGG | CGGAATTGTGCATTTACGTGAG |
| *omcB* | CGAGTTTATTTGCTAGCGGG | TCAACAACAACGTTACGAGC |
| IDO | CCTGACTTATGAGAACATGGACGT | ATACACCAGACCGTCTGATAGCTG |
| 18S rRNA | TCAAGAACGAAAGTCGGAGG | GGACATCTAAGGGCATCACA |
