# Supplementary figures and images for: Differential infection outcome of Chlamydia trachomatis in human blood monocytes and monocyte-derived dendritic cells
Source: BMC Microbiol. 2014 Aug 14;14:209. doi: 10.1186/s12866-014-0209-3 (PMC4236547; doi:10.1186/s12866-014-0209-3)

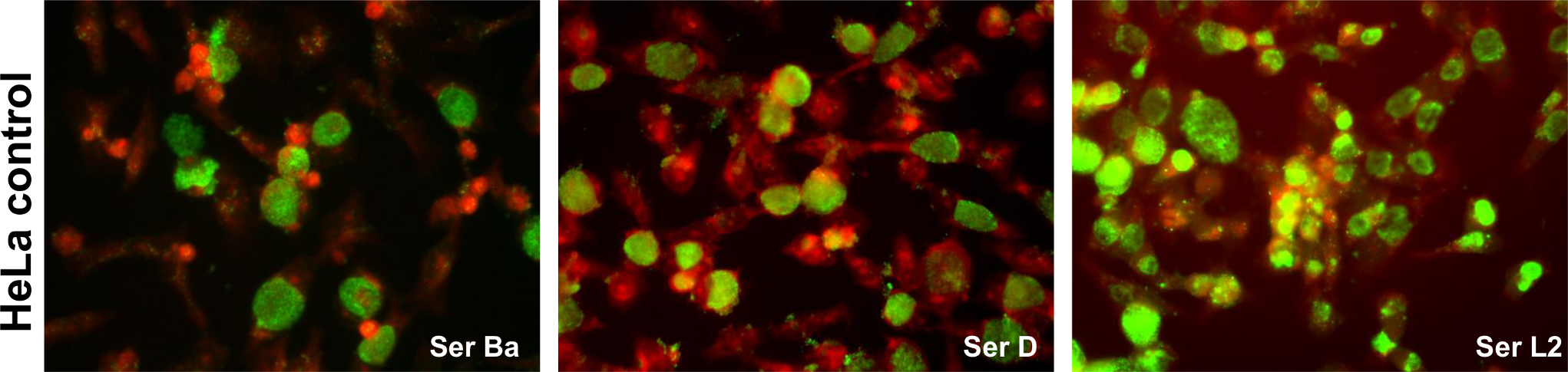

Supplement: Additional file 2: Figure S2. — Immunofluorescence microscopy of HeLa cells: HeLa cells were infected with C. trachomatis serovars Ba, D and L2 (MOI-3) for 2 days as positive control. Chlamydial inclusions (green) were stained with FITC conjugated anti-chlamydia LPS antibody and counterstained with Evans Blue. Pictures were taken at 40X magnification with Leica DMLB. The figures are representative of 3 independent experiments. [file s12866-014-0209-3-S2.tiff]

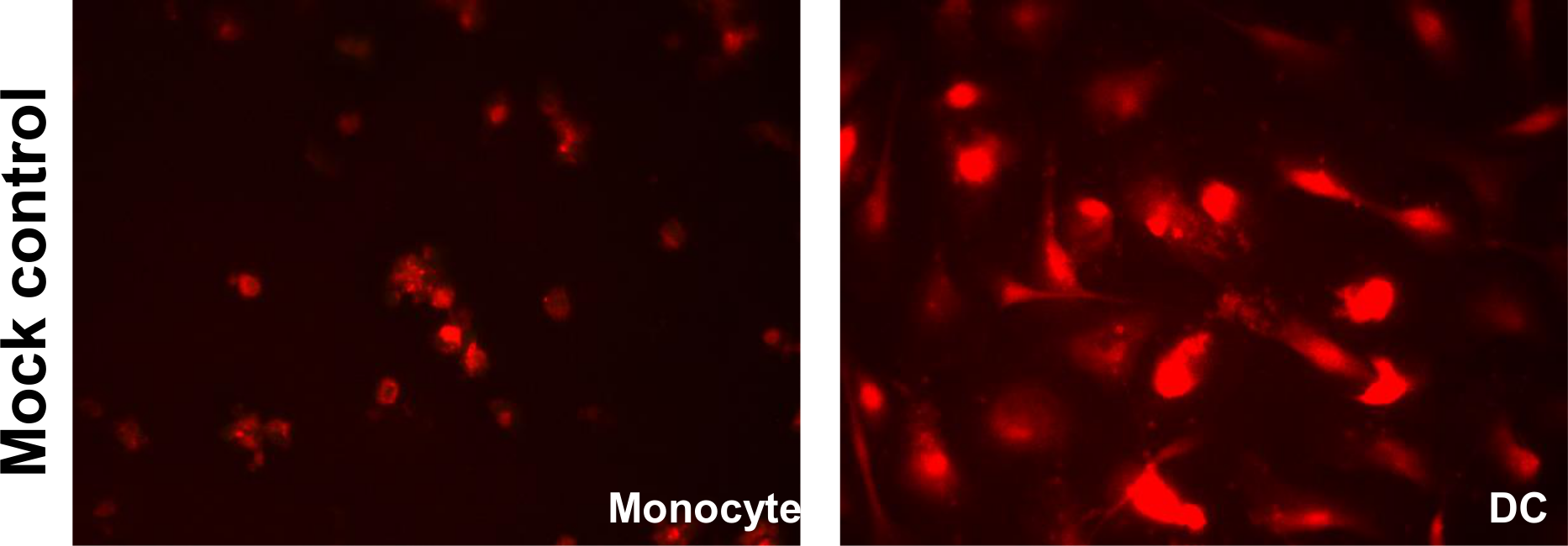

Supplement: Additional file 3: Figure S3. — Immunofluorescence microscopy of mock-infected monocytes and monocyte-derived DCs: Monocytes and monocyte-derived DCs were infected with mock control for 2 days. Chlamydial inclusions (green) were stained with FITC conjugated anti-chlamydia LPS antibody and counterstained with Evans Blue. Pictures were taken at 40X magnification with Leica DMLB. The figures are representative of 3 independent experiments. [file s12866-014-0209-3-S3.tiff]

**Figure S4** **TNF**

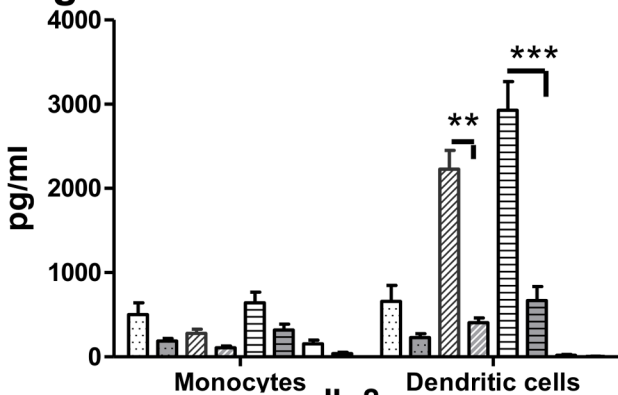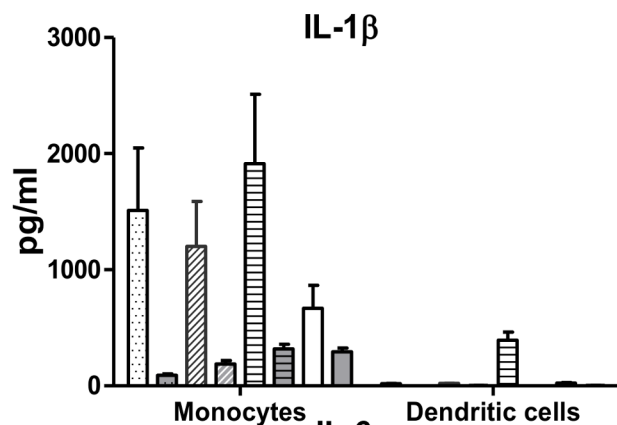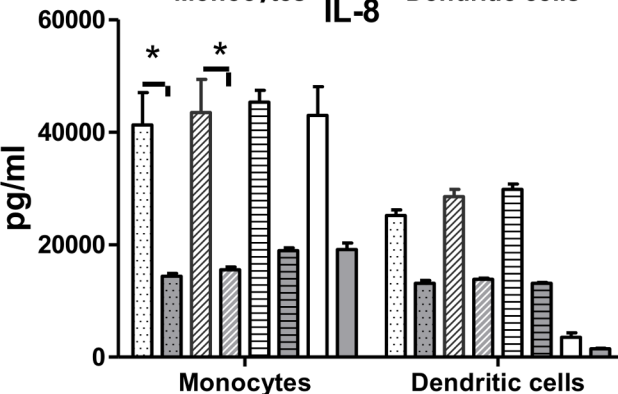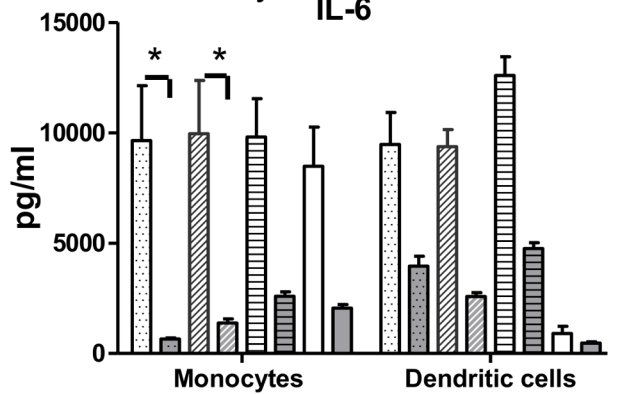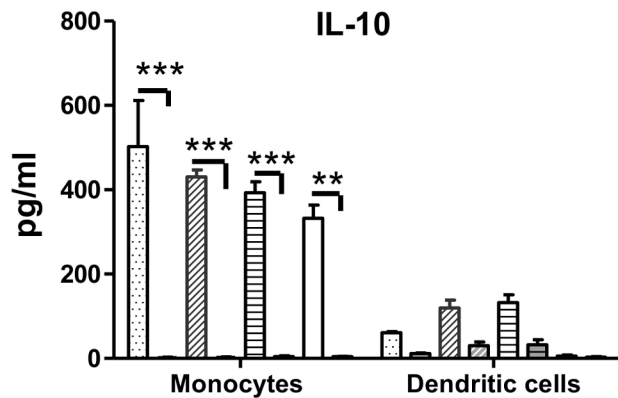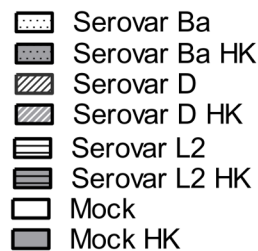

Supplement: Additional file 4: Figure S4. — Effect of heat-killed chlamydia in cytokine induction within infected monocytes and monocyte-derived DCs: Monocytes and monocyte-derived DCs were infected with live and heat-killed EBs of C. trachomatis serovars Ba, D and L2 (MOI-3) and mock control. Supernatants were collected 1 day post infection and the concentration of the different cytokines IL-1β, TNF, IL-6, IL-8 and IL-10 were determined by using the kit Cytometric Bead Array. The concentration is reported as pg/ml. The mean of 3 independent experiments is shown and each experiment is pool of 2 donors. ***P < 0.001, **P < 0.01, *P < 0.05. [file s12866-014-0209-3-S4.pdf]
